# Targeting Chondroitin Sulphate Synthase 1 (Chsy1) Promotes Axon Growth Following Neurorrhaphy by Suppressing Versican Accumulation

**DOI:** 10.3390/molecules28093742

**Published:** 2023-04-26

**Authors:** Chiung-Hui Liu, Ying-Jui Ho, Che-Yu Wang, Chao-Chun Hsu, Yin-Hung Chu, Min-Yen Hsu, Shiu-Jau Chen, Wen-Chuan Hsiao, Wen-Chieh Liao

**Affiliations:** 1 Ph.D. Program in Tissue Engineering and Regenerative Medicine, College of Medicine, National Chung Hsing University, Taichung 402202, Taiwan; chiunghui.liu@gmail.com (C.-H.L.);; 2 Department of Post-Baccalaureate Medicine, College of Medicine, National Chung Hsing University, Taichung 402202, Taiwan; 3 Department of Psychology, Chung Shan Medical University, No. 110, Sec. 1, Jianguo N. Rd., Taichung 402306, Taiwan; 4 School of Medicine, Chung Shan Medical University, Taichung 402306, Taiwan; 5 Department of Ophthalmology, Chung Shan Medical University Hospital, Taichung 402306, Taiwan; 6 Department of Medicine, MacKay Medical College, New Taipei City 252005, Taiwan; chenshiujau@gmail.com; 7 Department of Neurosurgery, MacKay Memorial Hospital, New Taipei City 251020, Taiwan

**Keywords:** Chsy1, versican, nerve regeneration, end-to-side neurorrhaphy, peripheral nerve injury, Schwann cells

## Abstract

Versican is a chondroitin sulfate proteoglycan (CSPG), which deposits in perineurium as a physical barrier and prevents the growth of axons out of the fascial boundary. Several studies have indicated that the chondroitin sulfate (CS) chains on versican have several possible functions beyond the physical barrier, including the ability to stabilize versican core protein in the extracellular matrix. As chondroitin sulfate synthase 1 (Chsy1) is a crucial enzyme for CS elongation, we hypothesized that in vivo knockdown of Chsy1 at peripheral nerve lesion site may decrease CS and versican accumulation, and result in accelerating neurite regeneration. In the present study, end-to-side neurorrhaphy (ESN) in Wistar rats was used as an in vivo model of peripheral nerve injury to evaluate nerve regeneration after surgical intervention. The distribution and expression of versican and Chsy1 in regenerating axons after ESN was studied using confocal microscopy and western blotting. Chsy1 was silenced at the nerve lesion (surgical) site using in vivo siRNA transfection. The results indicated that Chsy1 was successfully silenced in nerve tissue, and its downregulation was associated with functional recovery of compound muscle action potential. Silencing of Chsy1 also decreased the accumulation of versican core protein, suggesting that transient treating of Chsy1-siRNA may be an alternative and an effective strategy to promote injured peripheral nerve regeneration.

## 1. Introduction

Brachial plexus injury is a severe peripheral nerve trauma that results in functional damage and physical disability. In cases of peripheral nerve injury (PNI), end-to-side neurorrhaphy (ESN) surgery is performed to preserve the function of the donor nerve and the muscles it innervates, that ensure recovery of the injured nerve [1,2]. Neurorrhaphy facilitates the recipient nerve conduit to regenerate in new microenvironment, where there is mediated axon outgrowth and Schwann cell migration. Meanwhile, the accumulation of proteoglycans (PGs) at lesion tissue is known to play negative roles that hamper the regeneration process [3,4].

PGs are core proteins decorated with one to several linear glycosaminoglycan (GAG) side chains, which are one of the major components of the extracellular matrix (ECM). The PG bearing chondroitin sulfate (CS), a type of GAG chain, is defined as chondroitin sulfate proteoglycan (CSPG) [5]. CSPGs are considered to create a physical barrier that reduces neuronal plastic potential and consolidates myelinated fiber tracts. In particular, CSPGs of the lectican family are represented by aggrecan, brevican, neurocan, and versican [6,7,8], which are highly glycanated and abundantly distributed in the developing nervous system [9]. It is well known that CS is one of the major components of the glial scar, which prevents nerve regeneration in both the central nervous system (CNS) and the peripheral nerve system (PNS). Thus, methods to inhibit or discompose CSPG accumulation provide opportunities to improve nerve tissue regeneration [10,11].

The CSPG versican occurs in five isoforms (V0, V1, V2, V3, and V4), all of which include an N-terminal G1 domain, and a C-terminus containing a selectin-like (G3) domain (containing two EGF-like motifs) [8,12]. Versican V0 and V1 contain GAG-β domain and CS chains. Both versican V0 and V1 participate in guiding the outgrowth of peripheral axons during the PNS development [13,14]. In mature nerve system, versican V1 is present in the perineurium and in a subset of small basal lamina clusters within the endoneurium. Versican V2 has a GAG-α domain which widely presents in the CNS, whereas the smaller V3 isoforms lack both the GAG-α and GAG-β domains [9]. In the nerve lesion site, however, excessive versican presents in injured tissue and acts as an inhibitory barrier that blocks axonal outgrowth, which forces advancing axons into their designated trajectories during the initial stages of PNS formation [15].

The biosynthesis of CS chains on versican initiated from *N*-acetylgalactosamine (GalNAc) linking to a tetrasaccharide structure on core protein by CSGALNACT1 or CSGALNACT2. Next, the CS chain elongation is catalyzed by a group of β1–3 glucuronosyltransferase and β1–4 N-acetylgalactosaminyltransferase bi-functional enzymes (Chsy1, CHPF, CHPF2, and CHSY3) [16]. These CS chains are usually further modified by sulfurtransferase and CS epimerase at various sites. Thus, one CS chain may bear various modifications depending on the spectrotemporal expression of the polymerization and modification enzymes. These enzymes are also broadly distributed in nerve tissues, such as Chsy1, which are produced by neurons and astrocytes of the CNS as well as the Schwann cells of the PNS [10,17], and chondroitin 6-*O*-sulfated subunits are found in the basal lamina of the endoneurium [18].

Our previous studies indicated Chsy1 is a crucial enzyme governing CS formation in nerve tissue [19], and it constructs CS backbone for further modifications in peripheral neurons and nerve tissue [20]. This study focused on the expression and functions of Chsy1 during nerve regeneration. Furthermore, the correlation between nerve reconstruction and versican accumulation also be analyzed at early and late time points after ESN. We evaluated the effects of in vivo silencing Chsy1 on axonal outgrowth and versican distribution in ESN rats. Taken together, our results indicated that Chsy1-siRNA treatment modulated versican expression in regenerative nerve tissues, and effectively promoted axonal regeneration.

## 2. Results

### 2.1. Expression of Chsy1 in the Regenerating Axon after End-to-Side Neurorrhaphy

**Figure 1:**
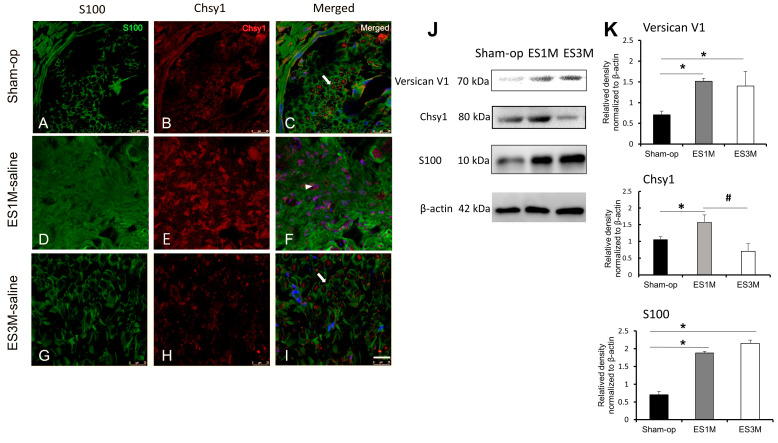
Confocal photomicrographs of the distribution of S100 and Chsy1 in regenerating axons at one and three months following ESN. Arrow indicated S100-positive cells. Arrow head indicated distribution of Chsy1.The recipient nerve tissue (distal end of McN) was immunostained with anti-S100 antibody (green) and anti-Chsy1 antibody (red) in the sham operation group (**A**–**C**), ES1M-saline group (**D**–**F**), and ES3M-saline group (**G**–**I**). After labeling the sections, nuclei DNA were counterstained with Hoechst33342 (blue). S100-positive Schwann cell and Chsy1 expression appear in the regenerating axons of ES1M group. Scale bar = 25 μm. Immunoblots (**J**) and histogram (**K**) showing versican V1, Chsy1, and S100 expressions in the nerve tissues of sham-operated and 1- to 3-month ESN rats. β-actin was used as a loading control. Data are present as mean ± SD from three independent experiments. * *p* < 0.05, compared with the sham operation group. ^#^ *p* < 0.05, compared with the ES1M si-ctrl group.

Schwann cells provide critical cell population support for axon outgrowth and myelination during peripheral nerve regeneration. To examine the distribution of Schwann cells and their expression of Chsy1 in the ESN model, the distal ends of the regenerating axons were immunostained with anti-S100 and anti-Chsy1 antibodies after surgery. Confocal photomicrographs revealed that Chsy1 was mainly present in the axons of the sham-operated group (Figure 1A–C). After one month of ESN, S100-positive cells and Chsy1 expression both showed an increase. Notably, Chsy1 was strongly expressed in the proliferative Schwann cells of the ES1M group compared to the sham operation group (Figure 1D–F). After three months, the Chsy1 expression significantly decreased in the Schwann cells and was present in the regenerating axons (Figure 1G–I). Western blots of the dissected nerve tissue confirmed the relative expression of Chsy1 during the regeneration (Figure 1J,K). In addition, the expression of core protein of versican also increased in ESN tissue one month after surgery. These results suggest that both versican and the key CS synthase, Chsy1, are increased at an early phase of peripheral nerve regeneration.

### 2.2. In Vivo Knockdown of Chsy1 after End-to-Side Neurorrhaphy

**Figure 2:**
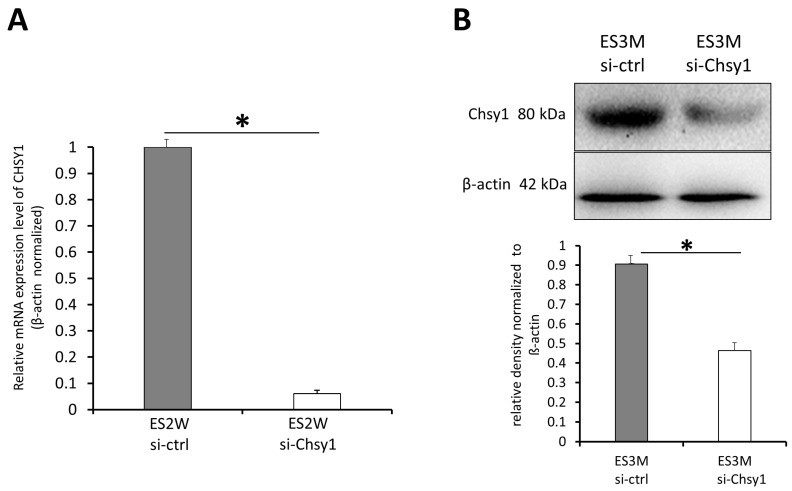
qPCR of the mRNA expression levels of Chsy1 in the recipient nerve tissue two weeks after the siRNA treatment. (**A**) Quantification of the qPCR analysis. The level of Chsy1 mRNA was decreased after 2 weeks after siRNA treatment (ES2W). * *p* < 0.05; as compared to that of the si-ctrl group. Values are presented as mean ± standard deviation. (**B**) Immunoblot showing Chsy1 expressions of the nerve tissues was lower in the si-Chsy1 group than in the si-ctrl group three months after siRNA treatment (ES3M). β-actin was used as a loading control. Data are present as mean ± SD from three independent experiments. * *p* <0.05, compared to that of the ES3M si-ctrl group.

Upregulation of Chsy1 may increase CS accumulation and inhibit nerve regeneration. Thus, we developed a Chsy1-specific siRNA treatment to silence Chsy1 in our ESN model. To determine the effect of siRNA on Chsy1, the in vivo knockdown efficiency of *Accell* siRNA in the nerve tissue was microinjected in the recipient nerve tissue and measured by qPCR. The qPCR results indicated that mRNA expression decreased in the si-Chsy1 group compared with the si-ctrl group at two weeks after siRNA treatment. Quantitative analysis revealed that the Chsy1 knockdown efficiency is 95% (Figure 2A). We also confirmed the decrease of Chsy1 protein in ES3M tissue by western blotting (Figure 2B), indicating this treatment is suitable for transient silence of Chsy1 in lesion tissue.

### 2.3. Effects of Chsy1-Silencing on Functional Recovery

**Figure 3:**
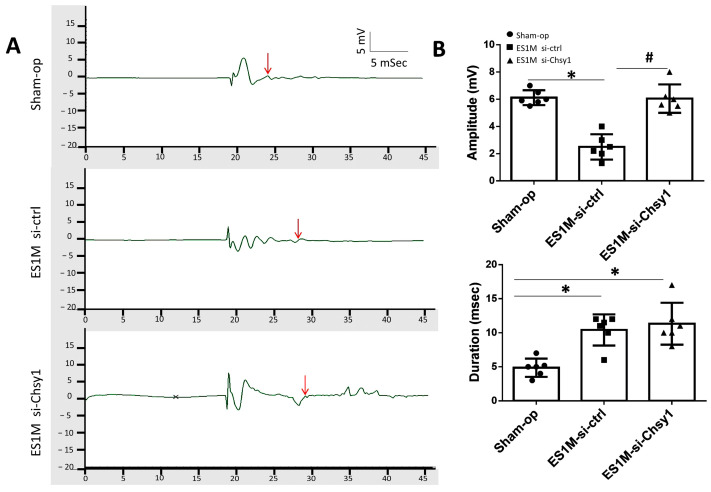
Compound muscle action potentials (CMAP) analyses for evaluating the functional recovery of ESN rats. (**A**) The recovery of CMAPs after ESN. The responses were recorded from the biceps brachii muscle upon activation of the nerve. The representative responses recorded from the sham operation group (upper row), ES1M si-ctrl group (middle row), and ES1M si-Chsy1 group (lower row) are illustrated. The arrow indicates the end of the duration. Stimuli at moderate (5 mA) strengths were applied to the nerve above the neurorrhaphy site. Bar-dot plots (**B**) show the averages of amplitude and duration, respectively. *n* = 6 for each group. Values are the mean ± standard deviation. * *p* < 0.05 compared to that of the sham-operated value. ^#^ *p* < 0.05 compared to the corresponding ES1M rats.

To confirm the relationship between Chsy1 and nerve conduction during nerve regeneration, we further examined the action potential of regenerating nerves using the compound muscle action potential (CMAP) analysis. The responses were recorded from the biceps brachii muscle upon nerve activation. According to the electrophysiological recordings, the ES1M si-ctrl group generated low amplitude and long-duration action potentials in comparison to the sham operation group. Nevertheless, the ES1M si-Chsy1 group demonstrated that motor function was rehabilitated and generated CMAPs with amplitudes similar to the sham operation group (6.00 ± 0.05 mV in sham operation, 6.20 ± 0.10 mV in ES1M si-Chsy1). Furthermore, the duration of ES1M si-ctrl (29.66 ± 1.52 msec) and ES1M si-Chsy1 (30.0 ± 0.81 msec) was significant (* *p* < 0.05) compared to the sham operation group (25.33 ± 0.57 msec) (Figure 3A,B). 

### 2.4. Effects of Chsy1-Silencing on Axonal Markers and Versican Expression

**Figure 4:**
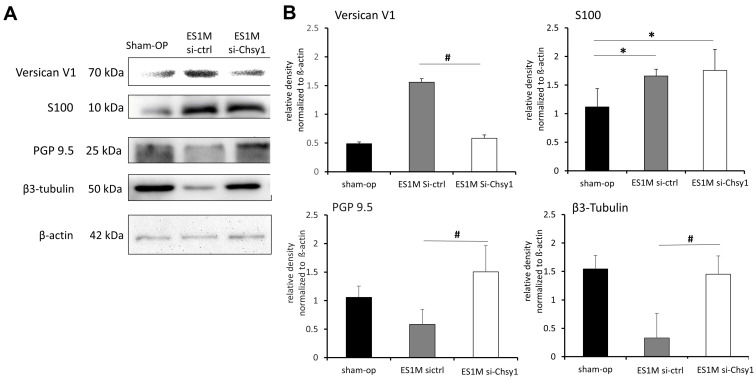
Protein expressions in the recipient nerve tissue one month after the siRNA treatment. (**A**) Western blots showing versican V1 expression and S100, PGP9.5, and β3-tubulin distribution in the sham operation, ES1M si-ctrl, and ES1M si-Chsy1 groups. (**B**) Histogram showing the expression of each protein in the sham-op, ES1M si-ctrl, and ES1M si-Chsy1 groups. β-Actin was used as a loading control. Data are present as mean ± standard deviation from three independent experiments. * *p* < 0.05, compared to the sham operation group. ^#^ *p* < 0.05, compared to the ES1M si-ctrl group.

The effects of Chsy1-silencing on the expression of versican V1 and nerve regeneration markers were further analyzed by western blots. Our results indicate that versican V1 was significantly decreased in the si-Chsy1 group one month after ESN (Figure 4A,B). Meanwhile, the protein levels of the axon markers, PGP9.5 and β3-tubulin, were obviously increased when Chsy1 was silenced by siRNA. In addition, the expression of S100 was unaffected. The sham operation tissue was analyzed in parallel as a healthy tissue control (Figure 4A,B). These results suggest that silencing the expression of Chsy1 in ESN tissue may cause versican downregulation and promote the recovery of nerve tissue.

### 2.5. Morphological Studies of Nerve Regeneration after siRNA Treatment

**Figure 5:**
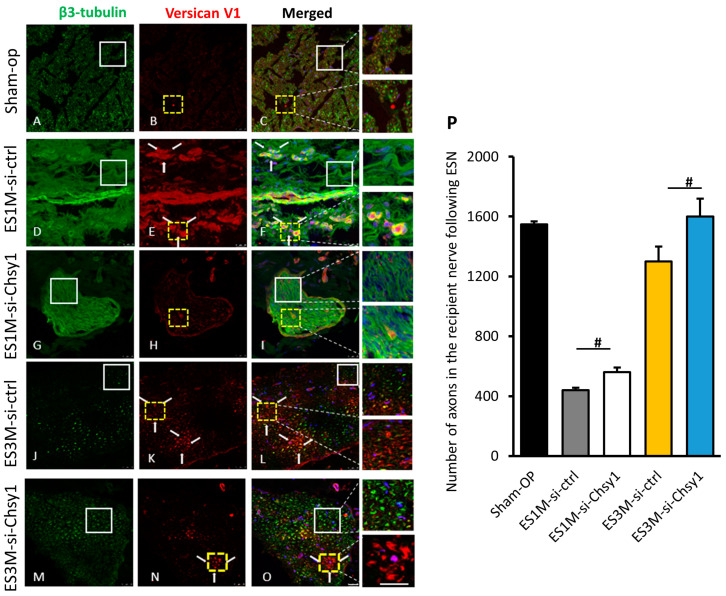
Distribution of β3-tubulin and versican V1 in the regenerating nerve tissue after siRNA treatment. The confocal photomicrographs show the distribution of β3-tubulin and versican V1 expression in the regenerating axons. The recipient nerve tissue (distal end of McN) was immunostained with anti-β3-tubulin (green) and anti-versican V1 (red) in 1-/3- month ESN after siRNA injection. After labeling the sections, nuclei DNA were counterstained with Hoechst33342 (blue). There is both β3-tubulin and versican expression in the axons of the sham operation (**A**–**C**), ES1M si-ctrl (**D**–**F**), ES1M si-Chsy1 (**G**–**I**), ES3M si-ctrl (**J**–**L**), and ES3M si-Chsy1 (**M**–**O**) groups. Amplified images are shown on the right. Staining of β3-tubulin is shown in the serial sections of the recipient nerve to indicate the location of the regenerating axon (left panel). Triple arrows indicate a cluster of versican V1 accumulation that hindered the regenerating axon path (middle panel). Note that for the ES3M si-ctrl group (**L**); rectangle and yellow dotted line box) and the ES3M si-Chsy1 group (**O**); rectangle and yellow dotted line box), the majority of β3-tubulin-positive nerve fibers were not co-localized with versican V1. Scale bar = 30 μm. Histogram (**P**) shows an average number of sprouting axons in each group 1–3 months after siRNA treatment. Data are present as mean ± SD; ^#^ *p* < 0.05, compared with the ES1M si-ctrl group.

Confocal microscopy was used to analyze the regenerating nerve tissue after siRNA treatments. Versican V1 and β3-tubulin were double stained on ESN tissue at one month and three months after surgery. The sham operation tissue was taken as a healthy nerve tissue control, where the versican expression is low and axons (β3-tubulin) were condensed and evenly distributed in the nerve cross section (Figure 5A–C). In the one-month ESN nerve sections, Chsy1 siRNA treatment revealed more arranged and thicker axon fibers than the control siRNA group, and the versican expression was decreased within the axon and surrounding tissue (Figure 5D–I). After three months recovery, more condensed axons were observed in Chsy1-siRNA treating tissue, compared with the control group. Additionally, there was less versican protein clustering within the Chsy1-siRNA treating nerve bundle (Figure 5J–O). Counting the absolute number of β3-tubulin positive axon numbers in nerve cross-sections indicated the axon number was slightly but significantly increased in the Chsy1 siRNA treating tissue (Figure 5P).

## 3. Discussion

The present study is the first to report that in vivo knockdown of Chsy1 by local injection of Chsy1-specific siRNA promotes axonal regeneration after ESN, and the decrease of versican may be involved in this process. We demonstrated that Chsy1 and versican V1 expression in the extracellular matrix were acutely upregulated one month after ESN, while the level of Chsy1 declined three months after surgery. It has been reported that versican V1, as an ECM component, modulates the regeneration and inflammation of PNS tissue by regulating Schwann cell activation [15,21]. These results suggest that versican could be a potential target in the control of axon regeneration and phenotypic changes associated with recovery progression. As mature versican is highly CS glycanated, we delivered an efficient pool combination of four Chsy1-specific siRNAs to inhibit CS polymerization on CSPGs, and consequently decreased vesical accumulation in the nerve tissue of ESN models.

The chemical-modified siRNA to allow for delivery without transfection reagents achieved potent gene silencing for more than two weeks after local delivery in the eyes, CNS, dorsal root ganglion, or tumors of the animal while using a low dose (2–1.5 nmol) to minimize side effects [22,23,24,25]. These results agree with our analysis in which nanomolar concentrations of siRNA were used to perform Chsy1 knockdown in vivo. Although the efficacy of siRNA in the peripheral nerve tissue was not stated in previous studies, we were able to achieve up to 50% efficiency of Chsy1 silencing, which succeeded in decreasing versican accumulation at the early recovery phase of ESN. Since the ES1M-si-Chsy1 group had more functioning motor axons than the ES1M-si-ctrl group connected to muscle fibers one month after surgery, the CMAP of the ES1M-si-Chsy1 group could generate the normal range of amplitude range. However, the immature regenerating fibers of the ES1M-si-Chsy1 and si-ctrl groups also had slow velocities due to the thin myelin thickness producing a more dispersed CMAP and a longer duration of the action potential.

The decrease of versican levels when silencing Chsy1 is in agreement with previous studies which showed that the loss of CS-GAG from the tissue leads to CSPG degradation, such as versican, and CD44 [26,27]. Our results also revealed that β3-tubulin-positive axons gradually reappeared in the distal end of the recipient nerve after Chsy1-siRNA treatment, which was associated with the loss of versican V1 accumulation. It is worth noting that, including versican, the CS on other CSPG may also be affected by Chsy1-siRNA treatment in our model. Indeed, several studies have proposed that enzyme degradation of CS or blocking CS bioactivities reveal positive as well as negative impacts on nerve regeneration. For instance, a recent study discovered CS-specific binding short peptides via a phage display peptide library, proving it can modulate neurite outgrowth [28]. Moreover, the excessive CS in the injured nerve tissue can be decomposed by a bacterial enzyme, chondroitinase ABC (ChABC) [29,30,31]. However, complete degradation of the inhibitory CS-GAGs through ChABC treatment may result in uncontrolled axon sprouting, which could lead to increased aberrant regeneration outside the perineurium. In addition, ChABC is thermally unstable at 37 °C, and its activity is lost within 1–3 days [18,32]. Repeated administration of the bacterial enzyme ChABC may cause an immune response and infection [32]. Our present study proposed that a transient Chsy1 silencing approach could partially attenuate the CS-GAG synthesis pathway and reduce CS inhibition in axon regeneration by decreasing excessive CSPG, such as versican V1. We supposed that the method could preserve the functions of core protein of these CSPGs, such as EGF-like motifs in the G3 domain of versican could function as signal ligands more easily when regenerating axons are accompanied by Schwann cells growing into a recipient nerve [12,33,34]. Thus, the Chsy1-siRNA approach may be more efficient and more accessible than other CS-blocking methods.

Our findings indicate that the si-Chsy1 group’s amplitude significantly differed from the si-ctrl group, but there was no significant difference in the duration of CMAP. Since the ES1M-si-Chsy1 group has more functioning motor axons than the ES1M-si-ctrl group connected to muscle fibers, the CMAP of the ES1M-si-Chsy1 group could generate the normal range of amplitude. However, the immature regenerating fibers of the si-Chsy1 and si-ctrl groups one month after surgery also had slow nerve conduction velocities due to the thin myelin thickness producing a more dispersed CMAP and a longer duration action potential.

The S100 expression in the versican-rich regenerating nerves of the si-ctrl and si-Chsy1 groups was much higher than that in the sham-operated group one month after ESN. siRNA treatment in ESN rats had no impact on Schwann cell expression. Previous studies have found that the versican G3 domain in epidermal growth factor receptor (EGFR) signaling is believed to be a vital element of a complex signaling network involved in cell proliferation and cell adhesion [35,36,37]. In agreement with the results of previous studies, our study observed that siRNA targeting the CS-GAG chain had dominant functions in axon sprouting, and the reserved versican V1 did not affect Schwann cell proliferation.

In conclusion, we demonstrated that manipulating GAG extension on CSPG using siRNA after ESN increased axonal sprouting, neural plasticity, and improved functional recovery. Silencing Chsy1 expression mediates neurite outgrowth and changes in axon distribution and versican V1 deposition. Modulating CS-GAG by Chsy1-siRNA provides new insight into the interactions between sprouting axons and a high CSPG-rich microenvironment. Hence, targeting this interaction could shed light on the acceleration of nerve regeneration, such as the slowed recovery of ESN models.

## 4. Materials and Methods

### 4.1. Experimental Animals

Adult male Wistar rats weighing between 200 to 300 g (*n* = 42) purchased from the Laboratory Animal Center of the Chung Shan Medical University were used as experimental animals in this study. All experimental animals were housed under consistent conditions that provide sufficient space at a regular temperature range of 20–24 °C. All experimental procedures with surgical intervention were approved by the Laboratory Animal Center Authorities of the Chung Shan Medical University (IACUC Approval No 2174).

### 4.2. Surgical Procedures

The in vivo model of PNI was performed by means of end-to-side neurorrhaphy (ESN) in use. In brief, rats were anesthetized with an intraperitoneal injection of 7% chloral hydrate (Sigma-Aldrich, St. Louis, MO, USA). The rats were then placed on a prepared surgical microscope stage. To expose the left musculocutaneous nerve, an incision was made along the skin of the left arm until the axilla. The musculocutaneous nerve (McN) was then transected at the margin of the pectoralis major muscle. We sutured the distal stump of the McN nerve (recipient nerve) to the side of the ulnar nerve (donor nerve) with 10-0 nylon sutures (Ethilon, Edinburgh, UK) under a surgical microscope [38,39,40]. The wound was sutured with 5-0 silk, and the animals were monitored for 1–3 months after surgery. Immediately after ESN, all operated animals were divided into three groups. The sham operation group was classified as Group I by simply cutting the skin to expose the left musculocutaneous nerve without nerve injury. Subsequently, the wound was sutured immediately. Both Group II (si-ctrl group) and Group III (si-Chsy1 group) obtained end-to-side neurorrhaphy. Subsequently, Group II received non-targeting RNA treatment, and Group III (si-Chsy1 group) received si-Chsy1 therapy for 1 and 3 months, respectively (*n* = 6 in each).

### 4.3. siRNA Delivery in ESN Rats

Small interfering RNA to control non-targeting RNA was purchased from Dharmacon (Lafayette, CO, USA). Target siRNA for Chsy1 (from Dharmacon SMART pool combination of four siRNA) or non-target siRNA oligonucleotides (each 100 nM) were diluted with PBS separately for 5 min under sterile conditions. Group II and Group III were then administered intramuscularly with a target (T) or control non-target siRNA (si-ctrl) at 25 μg/kg weekly for one month after ESN operation (*n* = 6 in each). The relative gene expression in the nerve was administered with non-targeting siRNA versus siRNA targeting Chsy1, measured by RT-qPCR (14 days post injection).

### 4.4. RNA Isolation and Quantitative Real-Time Polymerase Chain Reaction (qRT-PCR)

Total RNA was extracted from the nerve tissue of ESN rats using the Trizol reagent, according to the manufacturer’s protocol (Invitrogen, Carlsbad, CA, USA). All RNA samples were digested with DNase I before RT-PCR. Quantitative PCR system Mx3000P (Stratagene, CA, USA) and Brilliant SYBR Green qPCR Master Mix (Stratagene, CA, USA) were used to analyze gene expression in Wistar rats according to the manufacturer’s protocol. β-actin was used as an internal control. The primers were as follows: Chsy1 (Rattus norvegicus; Accession No. NM_001106268) forward primer 5′-ctcgctgacagaatcaacca-3′ and reverse primer 5′-tcccatgaacctcacaaaca-3′, β-actin (Rattus norvegicus; Accession: NM_031144.3) forward primer 5′-CTGGCACCCAGCACAATG-3′ and reverse primer 5′-AGCGAGGCCAGGATGGA-3′. Relative mRNA levels were calculated based on the cycle threshold values, corrected for β-actin expression, according to the equation: ΔΔCT = ΔCT (a target sample) − ΔCT (a reference sample) [41]. All experiments were performed in triplicate.

### 4.5. Compound Muscle Action Potential Recording

To monitor the functional status of a motor unit pool in ESN rats, the measurement of compound muscle action potentials (CMAPs) from the brachial plexus nerve using needle electrodes was applied here. CMAPs in the repaired nerve and target muscle were monitored using a PowerLab electromyogram made by AD instrument (Sydney, Australia). For recording, a silver stimulating electrode was placed under the reconnection site and a silver recording electrode was inserted into the biceps brachii muscle at the middle arm. A distance of 10 mm between recording and stimulating sites was applied for CMAP recording in the recipient nerve. To provide a path for current, the rat’s tail was connected to a ground wire to complete the circuit. Next, a current of 5 mA with 0.2 ms square pulse at a repetition rate of 0.2 Hz was applied. Data were then recorded, digitized, and analyzed.

### 4.6. Immunofluorescence Stain

For S100, Chsy1, versican V1 and β3-tubulin immunofluorescence double staining, the collected tissue sections were first put in a blocking solution (0.1% triton X-100, 3% normal goat serum, and 2% bovine serum albumi). All sections were incubated for 60 min at RT. After the protein blocking step, the sections were incubated in rabbit polyclonal anti-S100 antibody (1:1000, Taiclone Biotech Corp, Taipei, Taiwan), rabbit polyclonal anti-Chsy1 antibody (1:1000, Origene, Rockville, MD, USA), rabbit polyclonal anti-versicanV 1 antibody (1:500, Invitrogen, ThermoFisher, Waltham, MA, USA), and rabbit polyclonal anti-β3-tubulin antibody (1:1000, Proteintech, Manchester, UK), with the blocking solution for 24 h at 4 °C. After washing 3 × 15 min each in PBS at room temperature, the sections were further incubated with Alexa Fluor anti-mouse IgG (1:200, Jackson Immuno-Research, West Grove, PA, USA) and Cy3-conjugated anti-rabbit IgG (1:200, Jackson Immuno-Research, West Grove, PA, USA) to observe S100, Chsy1, versican V1 and β3-tubulin, respectively. All mounted sections were examined and photomicrographed under a confocal fluorescence microscope (SP5, Leica Microsystems, Wetzlar, Germany). The *Z*-stacked confocal images of the nerve were captured with a confocal microscope (TCS SP8) to analyze the S100, Chsy1, versican V1 and β3-tubulin distribution and recovery of the repaired nerve.

Each confocal fluorescence microscopy z-series scanning image was established using a stacked series of scans of a nerve section (25 μm in total thickness). The z-stack images were obtained from regenerating axons (5 μm optical slice thickness, 6 z-sections collected at 0.5-μm intervals). Furthermore, six cryo-sections per animal were analyzed in the nerve tissue 300 μm from the suture site. The number of β3-tubulin-positive axons was analyzed with a PC-based program (FREEMAN IM-AGE-PRO PLUS, Media Cybernetics, Silver Spring, MD, USA) from digital photographs of the sections.

### 4.7. Western Blotting

The musculocutaneous nerve tissue samples from different groups and time points after end-to-side neurorrhaphy were subjected to western blot analysis. Three tissue samples of distal McN removed from each group was first homogenized with Kaplan buffer (50 mM Tris buffer, pH = 7.4, 150 mM NaCl, 10% glycerol, 1% NP40, and a protease inhibitor cocktail) and then clarified by centrifugation. Then, equal amounts of solubilized proteins and cell lysates were separated on SDS-PAGE (8 & 10%) and electroblotted onto nitrocellulose membranes (Bio-Rad Laboratories, Hercules, CA, USA). The membranes were then blocked with 5% skim milk and probed with antibodies against versican V1 (1:500, Invitrogen, Thermo Fisher, Waltham, MA, USA), EGFR (1:1000, cell signaling), S100 (1:1000, Taiclone Biotech Corp), β-actin (1:10,000, BD Biosciences, San Jose, CA, USA), PGP9.5 (1:500, Abcam, Cambridge, UK), and β3-tubulin (1:1000, Proteintech, Manchester, UK) at 4 °C overnight. The membranes were then incubated with horseradish peroxidase-conjugated secondary antibodies (Bethyl Laboratories, Montgomery, TX, USA) at a dilution of 1:10,000 for 1 h at room temperature. The immunoreaction was detected using ECL kit (Millipore, Temecula, CA, USA)

### 4.8. Statistical Analysis

All quantitative data acquired from spectrometry, immunofluorescence, and immunoblotting in sham-operated and ESN rats were subjected to Prism 9 software (PRISM, GraphPad Software, San Diego, CA, USA) for analysis. Data were presented as the mean ± SD. *p* < 0.05 was considered statistically significant. A one-way ANOVA with a Bonferroni post hoc test was used to compare the level of each protein in the nerve tissues between the sham-op group, ES1M group, and ES3M group (Figure 1). Student *t*-test was used to compare the mean mRNA expression levels and protein levels from the ES1M-Chsy1 group and ES1M-Chsy1 group (Figure 2, Figure 4 and Figure 5). *p* < 0.05 is considered significant.

## Data Availability

The datasets used and/or analyzed during the current study are available from the corresponding author on reasonable request.

## Sample Availability

Samples of the compounds are available from the authors.
